# Naturopathic Interventions for Reduction of Perceived Pain in Patients Suffering from Arthritis: A Systematic Review and Meta-Analysis

**DOI:** 10.7759/cureus.54589

**Published:** 2024-02-20

**Authors:** Himel Mondal, Satyalakshmi Komarraju, Sathyanath D, Shrikanth Muralidharan

**Affiliations:** 1 Physiology, All India Institute of Medical Sciences, Deoghar, Deoghar, IND; 2 Naturopathy and Yoga, National Institute of Naturopathy, Pune, IND; 3 Research, National Institute of Naturopathy, Pune, IND

**Keywords:** life style, dietary supplements, physical therapy modalities, inflammation, arthralgia, osteoarthritis, rheumatoid, arthritis, naturopathy, chronic pain

## Abstract

Arthritis affects millions of lives with its pervasive effects on physical health and quality of life. Addressing the complexities of managing symptoms such as swelling, inflammation, and pain requires prolonged treatment. Naturopathy is a treatment method that enhances the body's innate ability to restore optimal health through a holistic approach including natural products and lifestyle modifications. This systematic review addresses the intersection of naturopathy and arthritis treatment to provide current evidence about its potential benefits. Four databases (PubMed, AYUSH Research Portal, Web of Science, and Google Scholar) were searched with the keywords "Naturopathy" AND "Arthritis". Randomized, non-randomized, and cross-over studies in English were included. Studies reporting perceived pain using a visual analogue scale (VAS) were selected for meta-analysis. A total of 15 studies were included in the systematic review. The studies were from Denmark, Egypt, France, Hungary, Israel, Italy, Spain, and Turkey, and the study periods ranged from 1992 to 2017. They suggested that naturopathic treatment modalities like exercise, mud compress, sand bath, or hydrotherapy may be used in addition to conventional modes of treatment for added benefit. There was a diversity of naturopathic treatment modalities and outcome evaluation methods. Most studies used mud compress or mud baths with reported improvement of symptoms. The meta-analysis of 10 studies (11 sets of data) showed a significant improvement in pain measured by VAS. The studies included in the review have a high level of heterogenicity. There is a need for more studies and uniform assessment methods with standardization of interventions for robust evidence. More clinical trials from countries where naturopathy is approved treatment modalities are needed.

## Introduction and background

Arthritis manifests as joint pain, swelling, stiffness, and decreased range of motion. It frequently leads to considerable functional impairment [1]. The effects of arthritis extend beyond the joints, impacting various aspects of an individual's life. Chronic pain is a hallmark feature that alters daily routines and diminishes the ability to engage in optimum levels of activities [2]. The relentless nature of arthritis pain not only affects the mobility of the patients but also takes a toll on their psychological and emotional well-being [3].

Effective management and treatment strategies for arthritis play a crucial role in mitigating these adverse effects. By addressing pain, preserving joint function, and improving overall well-being, healthcare interventions aim to enhance the quality of life for individuals living with arthritis [4,5]. Modern medicine treatment modalities may fail in many cases and a holistic approach including physical therapy and lifestyle modifications can be tried in conjunction with other treatment methods [6,7].

Among holistic and complementary therapeutic approaches, naturopathy has emerged as a promising avenue for the management of arthritis. Naturopathic treatments for arthritis often focus on incorporating various natural therapies including food modification (e.g., consumption of fresh fruits containing antioxidants, fish enriched with omega-3 fatty acids, foods containing calcium and vitamin D like yoghurt, and avoiding foods containing saturated fats, refined carbohydrates, sugar), massage, and compression with natural substances like mud. It emphasizes the body's innate ability to heal [8]. While some patients report relief, it's essential to note that scientific evidence supporting the efficacy of naturopathic treatments for arthritis is limited compared to conventional medical approaches [9]. There is no previous study that systematically evaluated the effect of naturopathic treatment for both upper limb and lower limb arthritis.

With this background, this systematic review aimed to review the literature on the treatment of arthritis by naturopathy therapies and a meta-analysis was done to find if the therapies help in the reduction of perceived pain after the therapy.

## Review

Methodology

The systematic review and meta-analysis were conducted in the Department of Physiology, All India Institute of Medical Sciences, Deoghar, India, and the Departments of Research and Naturopathy and Yoga, National Institute of Naturopathy, Pune, India. The study was conducted in three months from November 2023 to January 2024. The consensus meeting between the authors from two different settings was done via telephone and video calling.

A comprehensive search was carried out across four major databases namely PubMed, AYUSH Research Portal [10], Web of Science, and Google Scholar. Scopus and Embase could not be included due to the limitation of funds for accessing those databases.

The databases were searched with a combination of keywords: “Naturopathy” AND “Arthritis.” This specific combination aimed to target literature that specifically addressed the application of naturopathic interventions in the context of arthritis. For Google Scholar, due to the extensive number of results, we limited the selection to the first 100 (first 10 pages of search results with 10 results per page) articles retrieved. This approach aimed to strike a balance between inclusivity and practicality, considering the vastness of the Google Scholar database. In addition, cross-references were searched manually from studies.

Articles were included based on their relevance to the intersection of naturopathy and arthritis. Inclusion criteria were studies that explored the effectiveness of naturopathic treatments for arthritis, therapy applied for both upper limbs and lower limbs, and only clinical trials (randomized or non-randomized). Articles were excluded if they did not focus on the application of naturopathic interventions for arthritis or if they lacked sufficient methodological rigour. Studies that did not report complete data were omitted from the meta-analysis. Articles that were not in English were excluded due to the non-availability of language experts who could translate the literature effectively.

Relevant data from the selected articles were extracted systematically by two individuals and came to a consensus for final reporting. Synthesis involved a qualitative analysis of the findings, identifying patient characteristics, year of study, naturopathic interventions employed, duration of the treatment, outcome measurement, and summary of findings across the selected studies. Studies reporting the effect of therapy on perceived pain levels were included in the meta-analysis.

The systematic review was reported following Preferred Reporting Items for Systematic Reviews and Meta-Analyses (PRISMA). We used Microsoft Excel (Microsoft Corporation, Redmond, Washington, United States) for extracting and storing data and Review Manager 5.4.1 (The Cochrane Collaboration, 2020) for conducting the meta-analysis. For heterogeneity, used the criteria suggested by Dettori et al. [11].

Results

**Figure 1:**
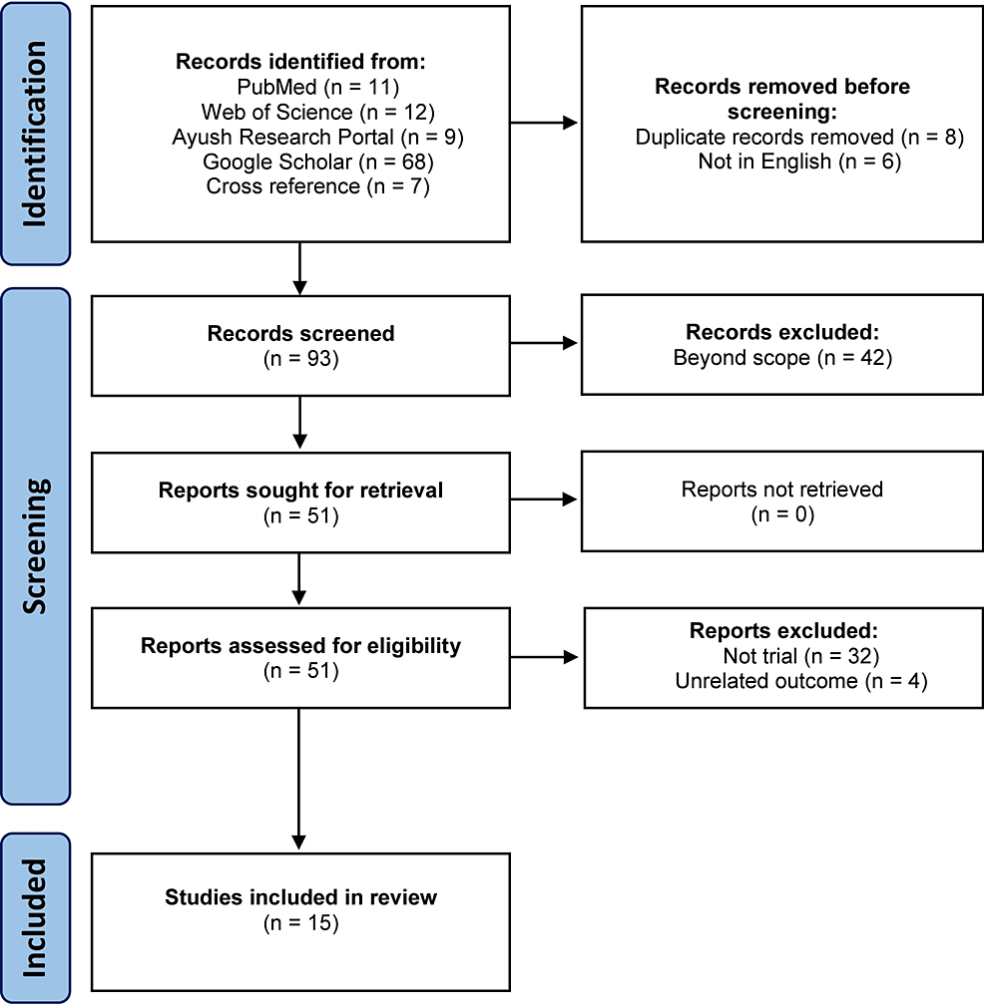
PRISMA flow chart showing the identification, screening, and inclusion steps PRISMA: Preferred Reporting Items for Systematic Reviews and Meta-Analyses

A total of 11 items from PubMed, 12 from Web of Science, nine from AYUSH Research Portal, 68 from Google Scholar were initially identified, and seven were obtained from cross-references. After removing duplicates, non-English studies, and excluding studies according to inclusion and exclusion criteria, a total of 15 studies were included in the final analysis. The PRISMA flow chart is shown in Figure 1.

The characteristics of the included studies with their major finding are summarized in Table 1.

**Table 1:** Characteristics and summary of the results obtained from the studies included in the systematic review *Studies included in the meta-analysis; **The study by Sukenik et al. did not report SD and hence only the value of mean is given VAS: visual analogue scale; WOMAC: Western Ontario and McMaster Universities Arthritis Index; SF-36: 36-Item Short Form Survey; HAQ: Health Assessment Questionnaire; W-TPS: total pain score (WOMAC subscore); W-TSS: total stiffness score (WOMAC subscore); W-TPFS: total physical function score (WOMAC subscore); HRQOL: health-related quality of life; EuroQoL-5D: EuroQol- 5 Dimension; OA: osteoarthritis

Author, year	Place	Sample in intervention (n), age (years) (mean±SD)	Sample in control (n), age (years) (mean±SD)	Region of body	Therapy in the intervention group	Therapy in the control group	Duration	Outcome	Summary of finding
Sukenik et al., 1992 [12]	Israel	14, 51.9^**^	14, 60.5^**^	Upper and lower	True mud pack therapy	Washed-out mud pack therapy	2 weeks	Morning stiffness, hand-grip strength, perceived disease activity, number of active joints, Ritchie index	Improvement of objective and subjective symptoms after therapy
Flusser et al., 2002* [13]	Israel	40, 64.7±7.9	18, 64.8±7.3	Lower	Mineral-rich mud pack therapy	Mineral-depleted mud pack therapy	3 weeks	Lequesne Index of severity of knee osteoarthritis, self-assessment of knee pain, VAS, range of movement, soft tissue swelling, effusion or crepitus	Significant reduction in knee pain
Codish et al., 2003* [14]	Israel	22, 57.5±9.8	23, 60.1±12.4	Upper	Mineral-rich mud compresses	Mineral-depleted compresses	3 weeks	Number of painful and swollen joints of both hands, VAS	Reduction of the number of painful and swollen joints, less perceived pain
Evcik et al., 2006* [15]	Turkey	25, 55± 8.7	25, 59.6 ± 9.2	Lower	Balneotherapy	Hot pack	2 week	VAS, WOMAC, Quality of life	Balneotherapy is an effective treatment for knee OA
Evcik et al., 2006* [15]	Turkey	25, 57.4 ± 9	25, 59.6 ± 9.2	Lower	Mud pack	Hot pack	2 week	VAS, WOMAC, Quality of life	Mud-pack therapy is an effective treatment for knee OA
Forestier et al., 2007 [16]	France	195, 63±9.1	187, 64.3±10.4	Lower	Spa therapy (massages, showers, mud and pool sessions) exercise	Exercise	3 weeks	VAS, WOMAC, quality of life	Minimal clinically important improvement, no improvement in quality of life
Lund et al., 2009 [17]	Denmark	19, 73.1±9.4	19, 73.1±9.4	Lower	Stimulating massage of the muscle	Rest	10 min	Joint repositioning error	Massage has no beneficial effect on the sense of joint position
Fioravanti et al., 2010 [18]	Italy	40, 69.06 ±5.11	40, 71.3 ±4.91	Lower	Daily routine mud pack	Routine care	2 weeks	VAS, Lequesne Index, WOMAC, Arthritis Impact Measurement Scale	Positive effects on the painful symptomatology
Gungen et al., 2011* [19]	Turkey	23, 65.04 ± 7.11	21, 61.87 ± 6.73	Lower	Mud compress	Hot pack	2 weeks	VAS, range of motion, 15-m walking time, WOMAC, Nottingham Health Profile	Mud packs and hot packs both are effective in reducing symptoms
Sarsan et al., 2012* [20]	Turkey	15, 52.4 ± 5.2	12, 53.6 ± 8.0	Lower	Mature mud pack	Hot pack	2 weeks	VAS, WOMAC, 6 min walking distance, quality of life SF-36	A mud pack is more favorable than a hot pack
Antúnez et al., 2012* [21]	Spain	61, 69.13±5.60	60, 73.08± 8.90	Lower	Daily sessions of Peloids and routine drug therapy	Routine drug therapy	11 days	VAS, HRQOL	Mud therapy reduces pain and improves quality of life
Tefner et al., 2013 [22]	Hungary	27, 63.42 ± 8.86	26, 63.55 ± 9.53	Lower	Hot mud-pack therapy	Mud pack	2 weeks	WOMAC, EuroQoL-5D	Mud pack improves clinical parameters, quality of life, and reduce the need for medications
Fioravanti et al., 2014* [23]	Italy	49, 68.12±8.97	46, 69.70±10.32	Lower	Mud-bath therapy and regular treatment	Regular treatment	2 weeks	VAS, WOMAC, W-TPS, W-TSS,W-TPFS, serum adiponectin, resistin and visfatin	Mud-bath therapy modifies serum levels of adiponectin and resistin, but not levels of visfatin
Allam et al., 2016* [24]	Egypt	15, 41.73 ± 8.95	15, 42.53 ± 9.36	Whole body	Siwan traditional therapy followed by a massage with olive oil	Traditional physical therapy	2 months	VAS, HAQ	Siwan therapy is more effective than traditional physical therapy
Pascarelli et al., 2016* [25]	Italy	53, 68.49 ± 9.01	50, 69.66 ± 11.1	Lower	Mud bath therapy with regular care	Regular care only	2 weeks	Global pain score, WOMAC, VAS	Beneficial to reduce pain and improve function
Giannitti et al., 2017* [26]	Italy	21, 69.52 ± 7.17	11, 69.36 ± 11.29	Lower	Mud-bath therapy in addition to the usual treatment	Usual treatment	2 weeks	VAS, WOMAC, microRNA expression	Balneotherapy can modify expression of some microRNAs involved in OA

The studies in the list from 1992 to 2017 are from Denmark, Egypt, France, Hungary, Israel, Italy, Spain, and Turkey [11-16]. The studies suggested that naturopathic treatment modalities like exercise, mud compress, sand bath, or hydrotherapy may be used in addition to conventional modes of treatment for added benefit.

**Figure 2:**
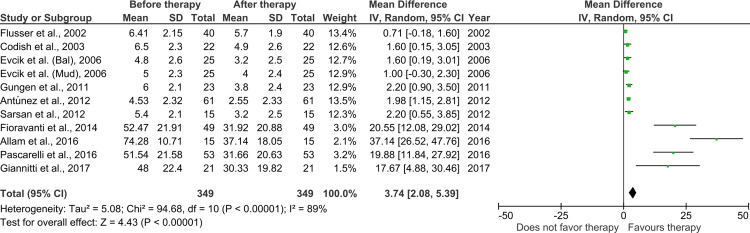
Comparison of pain measured by visual analogue scale in intervention group before and after therapy SD: standard deviation; IV: inverse variance; CI: confidence interval References: [13-16,19-26]

Among the studies, 10 studies (one study had two intervention groups; hence, a total of 11 sets of data) were selected for meta-analysis as they reported the mean and standard deviation of VAS for pain severity. The forest plot of the intervention groups in pre-therapy and post-therapy VAS scores is shown in Figure 2.

**Figure 3:**
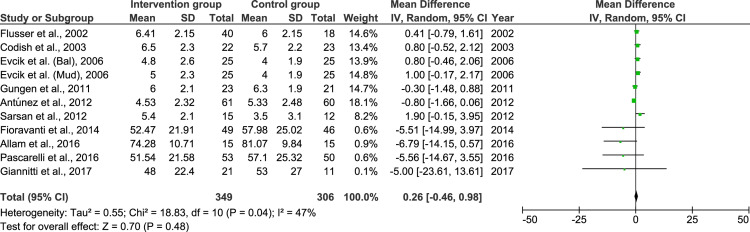
Comparison of pain measured by visual analogue scale in intervention and control group before therapy SD: standard deviation; IV: inverse variance; CI: confidence interval References: [13-16,19-26]

There was a significant decrease in perceived pain after the therapy in the intervention group. However, the studies were significantly heterogeneous. The perceived pain VAS score before the therapy in the intervention and control groups is shown in Figure 3.

**Figure 4:**
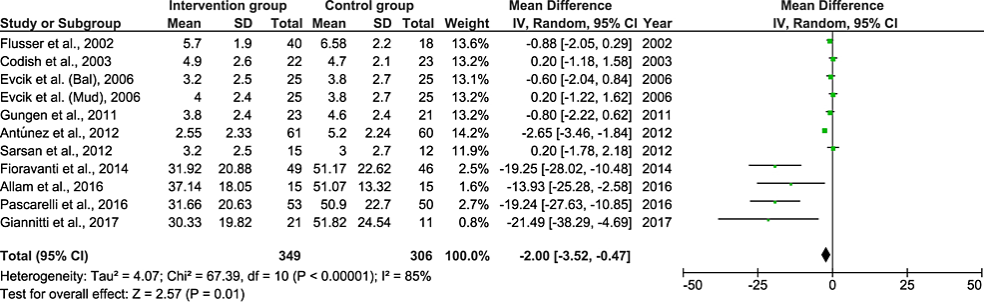
Comparison of pain measured by visual analogue scale in intervention and control group after therapy SD: standard deviation; IV: inverse variance; CI: confidence interval References: [13-16,19-26]

There was no evidence of a difference in the level of perceived pain in the intervention and control groups before starting the therapy. The studies had moderate heterogeneity. The pain score after the therapy in the intervention and control groups is shown in Figure 4.

There was significantly lower perceived pain in the intervention groups after the therapy when compared with controls. The heterogeneity was considerably high.

Discussion

The findings from the included studies suggest a potential role for naturopathy in the management of pain related to arthritis. The majority of the studies reported significant positive outcomes, including improvements in pain levels and joint function by reducing the swelling, redness, and number of painful joints. However, naturopathic therapy was used as an additional therapy to the routine treatment the patient was undergoing [27]. Hence, the therapies used in those trials do not suggest replacing the ongoing treatment but suggest additional measures that can help reduce the pain of arthritis. Diverse therapeutic approaches were used in the trials like Siwan therapy, involving sand bathing and olive oil massage, stimulating massage, spa therapy combined with exercises, and mineral-rich mud compresses and packs. Overall, the efficacy of these interventions was context-specific, emphasizing the need for tailored approaches in arthritis management.

Despite the overall positive results reported in the individual studies, it is crucial to acknowledge the heterogeneity in study designs and methodological quality among the included articles. Even for mud therapy, the mineral content in mud may be different according to countries and regions. Hence, uniform therapies were lacking. The variation in outcome measures, duration of interventions, and participant characteristics poses challenges in drawing definitive conclusions.

Among the studies, 10 studies were included in the meta-analysis and the result suggested significant improvement in perceived pain level as measured by VAS in the intervention group. This supports an earlier review by Hou et al. of mud therapy in knee osteoarthritis [28]. We combined the lower, upper, and whole-body therapies in the meta-analysis [14,24]. However, studies for upper limbs and the whole body are scarce and need further exploration. In addition to mud packs and other methods found in this review (Table 1), other naturopathic methods to control inflammation are yet to be explored [29,30].

When the therapy was started, baseline characteristics showed that the pain levels in both groups were similar (Figure 3). However, after treatment, the pain in the intervention groups significantly reduced (Figure 4). However, at this point, with different modes of naturopathic intervention, the synthesized evidence needs further exploration for a more robust and definite conclusion.

This systematic review has limitations. The restriction to English-language articles and the limited number of articles from Google Scholar may introduce selection bias. The risk of bias was not assessed. Furthermore, the keywords used in this study were selected to find the naturopathic therapies and studies that did not include the term "naturopathy" were excluded. For example, chiropractic therapy, which has similarities to naturopathy might not appear in the literature search [31]. Future research should aim for more extensive inclusion of diverse sources and languages. Additionally, well-designed randomized controlled trials are needed in countries where naturopathic treatments are approved therapy and are in the academic sphere to strengthen the evidence base and establish the sustainability of naturopathic interventions for arthritis [32].

## Conclusions

There was diversity of naturopathic treatment modalities and outcome evaluation methods. Mud packing was the most common method. Most studies showed improved disease severity when naturopathy is combined with other treatment methods. There was a significant improvement in perceived pain measured by VAS immediately after the therapy. There is a need for more studies with uniform therapy and assessment methods for robust evidence. More clinical trials from countries where naturopathy is approved treatment modalities are needed for more robust evidence.
